# A generic classification-based method for segmentation of nuclei in 3D images of early embryos

**DOI:** 10.1186/1471-2105-15-9

**Published:** 2014-01-14

**Authors:** Jaza Gul-Mohammed, Ignacio Arganda-Carreras, Philippe Andrey, Vincent Galy, Thomas Boudier

**Affiliations:** 1 Sorbonne Universités, UPMC Univ Paris 06, 4 place Jussieu, 75005 Paris, France; 2 CNRS-UMR7622, Université Pierre et Marie Curie, 4 place Jussieu, 75005 Paris, France; 3 INRA, UMR1318, Institut Jean-Pierre Bourgin, 78026 Versailles, France; 4 AgroParisTech, UMR1318, Institut Jean-Pierre Bourgin, 78026 Versailles, France; 5 University of Sulaimani, School of Engineering, 46001 Sulaimani, Iraq

**Keywords:** Image segmentation, Classification, Cell cycle, 3D, 4D, *C. elegans*, *Drosophila*, Development

## Abstract

**Background:**

Studying how individual cells spatially and temporally organize within the embryo is a fundamental issue in modern developmental biology to better understand the first stages of embryogenesis. In order to perform high-throughput analyses in three-dimensional microscopic images, it is essential to be able to automatically segment, classify and track cell nuclei. Many 3D/4D segmentation and tracking algorithms have been reported in the literature. Most of them are specific to particular models or acquisition systems and often require the fine tuning of parameters.

**Results:**

We present a new automatic algorithm to segment and simultaneously classify cell nuclei in 3D/4D images. Segmentation relies on training samples that are interactively provided by the user and on an iterative thresholding process. This algorithm can correctly segment nuclei even when they are touching, and remains effective under temporal and spatial intensity variations. The segmentation is coupled to a classification of nuclei according to cell cycle phases, allowing biologists to quantify the effect of genetic perturbations and drug treatments. Robust 3D geometrical shape descriptors are used as training features for classification. Segmentation and classification results of three complete datasets are presented. In our working dataset of the *Caenorhabditis elegans* embryo, only 21 nuclei out of 3,585 were not detected, the overall F-score for segmentation reached 0.99, and more than 95% of the nuclei were classified in the correct cell cycle phase. No merging of nuclei was found.

**Conclusion:**

We developed a novel generic algorithm for segmentation and classification in 3D images. The method, referred to as **A**daptive **G**eneric **I**terative **T**hresholding **A**lgorithm (AGITA), is freely available as an ImageJ plug-in.

## Background

Studying how individual cells spatially and temporally organize within the embryo is a fundamental issue in modern developmental biology to better understand the first stages of embryogenesis. Cell dynamics can be analyzed from three-dimensional (3D) images of labeled nuclei. To perform systematic studies and high-throughput analyses, automated methods that quantify nuclei over time and reconstruct cell lineages are required. For tracking purposes, detecting the centers of nuclei can be sufficient. However, for accurate geometrical and morphological analyses, a complete segmentation of nuclei is required. In addition, in cell-cycle studies, accurate classification of nuclei according to their cell-cycle stage is necessary.

For these reasons, many segmentation and/or tracking methods have been developed. Bao *et al.*[1] proposed local maxima detection followed by 3D spherical approximation for nuclei segmentation in *Caenorhabditis elegans*. Melani *et al.*[2] proposed the identification of nuclei using a spherical Hough transformation, and Soubies *et al.*[3] extended the procedure to the detection of ellipsoids. Segmentation of nuclei by 2D detections using difference of Gaussians and 3D reconstruction based on Bayesian features was used by Santella [4]. Another algorithm based on a Bayesian estimation framework for tracking was proposed by Carranza *et al.*, where the nuclei were detected using an h-dome transform [5]. Multiple level sets and background/foreground detection was proposed by Chinta *et al.*[6]. Most of these algorithms require fine-tuning of many parameters and are often only successful for dedicated applications and using specific acquisition systems or labeling protocols.

For the purpose of automation, a key issue is the objective determination of a correct intensity threshold for image segmentation. Due to imperfect imaging conditions and variations of fluorescence intensity with respect to time and depth, a unique global threshold generally fails to correctly detect nuclei. Therefore, the use of adaptive local thresholding is necessary to detect nuclei regardless of fluctuations in their intensity. The idea of testing multiple thresholds for each object and then deciding which threshold is the best is not new (see for example, [7]). Matas *et al.* presented MSER (Maximally Stable Extremal Regions), a method where the best threshold is the one that yields to minimal variations of the object surface. Keller *et al.* developed an algorithm based on this same idea to detect nuclei in developing zebrafish embryos imaged with digital scanned light sheet microscopy [8]. However, identifying and specifying *a priori* a suitable threshold selection criterion (i.e., yielding robust segmentation results) is a real challenge and may require the fine-tuning of several parameters.

Machine learning can be used to eliminate the need for an explicit threshold selection criterion. In supervised machine learning, samples of known categories are provided as input and the algorithm automatically finds a decision rule that most effectively separates the different classes. Arteta [9] uses a support vector machine to detect extremal non-overlapping regions in 2D images. Lin [10] proposed to use Bayesian models on 2D intensity and geometrical features. In this approach, a first segmentation is suggested and the user validates the correctly segmented objects that will be used for the training. Objects are then segmented using a classical watershed approach and the training is used to fuse separated regions.

In the present paper, we introduce a learning-based method to segment nuclei in 3D/4D images of early embryos. This work was developed to compensate for the lack of robust alternatives to segment our working dataset of the *C. elegans* embryo imaged with a spinning disk confocal microscope. Our method distinguishes itself from previous works in three ways. Firstly, we propose a segmentation technique that can be applied to different image acquisition conditions and various embryo models. Secondly, the number of parameters has been minimized to include just a few biological parameters. Lastly, our algorithm segments and classifies nuclei simultaneously, while other approaches first segment and then classify nuclei. Specifically, we chose the threshold leading to the object that is most similar to one in a set of training samples.

In this paper, we present the results of applying our novel algorithm to three different datasets containing embryo nuclei from *C. elegans*, *Drosophila* and 3D simulated data [11]. The algorithm has been implemented as an open-source plug-in for ImageJ [12] and is publicly available for download along with a tutorial and sample data [13].

## Methods

**Figure 1:**
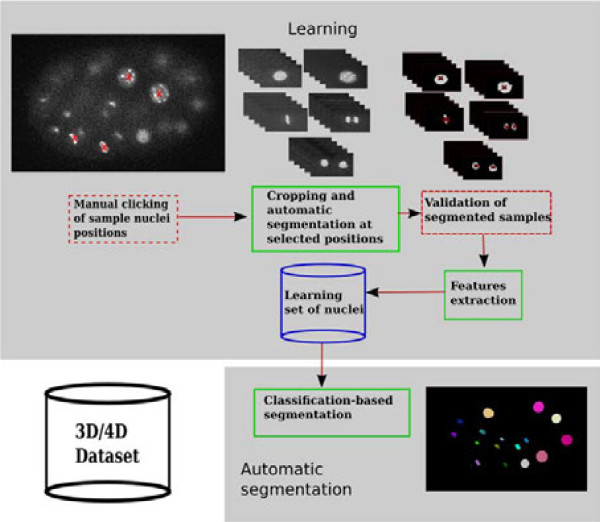
**Flowchart of the overall procedure.** The first step consists in training the program by learning sample nuclei. The user clicks on the approximate position of the nuclei in the image. Using an iterative thresholding algorithm, nuclei are automatically extracted around each clicked position. The user has to validate the segmented nuclei that will serve as training samples for the classifier. The second step is completely automatic and will segment, as well as classify, objects in the entire dataset.

The procedure is composed of three steps: filtering to reduce noise, supervised learning of user-selected training samples and, finally, classification-based segmentation of nuclei (Figure 1). The only biological parameters that must be supplied by the user are the approximate volumes of the nuclei for each cell cycle phase at the start and end time points of the dataset.

### Sample preparation of working dataset

The *C. elegans* early embryo dataset was acquired with a spinning-disk confocal microscope. The dataset is composed of a developing embryo from the 4-cell stage to the 90-cell stage with 160 time points. The signal consists in Histone-GFP expressed in the ovary and transmitted to the future embryo at the time of oocyte formation. At each cell division during embryo development, this maternal load is incorporated into the chromatin of an increasing number of cells, thus leading to a dilution of the fluorescence signal over time.

### Overview of the method

The main idea of the algorithm is to use machine learning to improve the segmentation results of cell nuclei. The learning step is an interactive process. The user first selects representative nucleus samples of each cell cycle by clicking on their locations inside the 3D or 4D dataset. The objects at the clicked positions are then segmented through an iterative thresholding procedure using the user-supplied volume estimates as a reference. After that, the user has to validate the proposed segmented nuclei, and 3D descriptors are computed from this set of validated nuclei. A predefined classifier is then trained to assign cell cycle phases to nuclei based on that set and those descriptors. The main joint segmentation/classification procedure is subsequently applied to all time points.

### Choosing and setting up the classifier

Before setting up the segmentation procedure based on classification, it was necessary to choose and set up a classifier. A first training set of manually-validated nuclei were extracted from the image using the procedure presented in the *Supervised learning* section below. Each object was represented by a feature vector of 3D descriptors calculated as described in the following section, and was assigned its corresponding cell cycle phase or *class* by the user. To find the most suitable learning algorithm, we made experiments across our datasets after dividing our samples into a training and a test set (66% and 33% of the data, respectively). For this purpose, we used the experimenter of the Waikato Environment for Knowledge Analysis (WEKA) suite [14] and the BIOCAT program [15]. We empirically found that a random forest classifier provided the best separation of our samples between the different cell-cycle phases. A random forest is an ensemble learning method based on the decisions of an arbitrary number of random decision trees [16]. 200 trees were used in our case. The decision trees are simple binary trees in which each node divides the set of samples based on the most differentiating feature at the given tree level. This way, the deeper we go down the tree, the better the samples are differentiated.

### Pre-processing

Filtering is a common pre-processing step to improve the results of subsequent segmentation by increasing the signal-to-noise ratio in the image. For this purpose, a classical 3D median-filter is used with a variable radius, which is proportional to the radius of the equivalent sphere for a given volume $R={\left(\frac{3V}{4\pi }\right)}^{\frac{1}{3}}$. This classical filter proved useful for noise reduction while preserving edges. Intermediate time point volumes are linearly interpolated from the minimum and maximum volumes supplied by the user at the learning step. Nuclei are large at early stages, so a large radius can be used to reduce noise and homogenize intensity signals inside the nuclei. Adapting the filter size ensures that late stage nuclei, which are smaller, are not removed.

In addition, in order to avoid unnecessarily long computing times when filtering with too large a radius, a limit of *R*=12 is proposed by the plug-in (12 pixels in the XY-plane; the radius used in Z takes the calibration of the image into account) and performs well in practice. The filtering step is optional and data can be pre-processed with another filtering sequence before segmentation, if appropriate.

### Supervised learning

In this preliminary step, classes are created in an interactive manner, and features of nuclei for each class are computed. For this purpose the user manually selects some samples of each class at different time points by clicking on the approximate position of the nucleus sample centers in the 3D or 4D image. A fixed-size box centered on the selected voxel is then cropped, and the extracted sub-volume goes through an iterative thresholding procedure similar to MSER. Briefly, for each threshold, the volume of the segmented object (closest to center of box) is computed and compared to the range of volumes given by the user. Once the object falls inside the interval of volumes, it is extracted. The same applies to all selected positions; each sample is therefore thresholded with a different threshold. The user then has to validate correctly segmented nuclei from the set of segmented nucleus samples.

### 3D shape descriptors

Finding a good set of features for machine-learning classifiers is an important task to obtain accurate classification. We tried to optimize the choice of descriptors so that they can be robustly used in different contexts of acquisition and species. Among the 3D descriptors available, we first eliminated intensity-based descriptors since intensity may vary between nuclei and between acquisitions. Secondly, we discarded descriptors that are size-dependent such as volumes or surfaces, since nuclei in the same cell-cycle phase (e.g., interphase) may have variable sizes, ranging from very large at the first time points to very small at the subsequent time points. We therefore focused on ten shape descriptors that are described below.

#### Compactness and sphericity

These factors describe how far the object is from a perfect sphere. These two descriptors are related and can be estimated from a normalized surface-to-volume ratio. Their values vary between 0 and 1 for a perfect sphere. These parameters are popular for evaluating morphological changes in biological entities [17].

(1) $$\text{Compactness}=\frac{36\pi V^{2}}{S^{3}},\text{Sphericity}=C^{\frac{1}{3}}$$

where *V* is the volume of the object (using calibrated voxel sizes in *X*, *Y*, and *Z*).*S* is the surface of the object (using calibrated voxel sizes in *X*, *Y*, and *Z*).

#### Ellipsoid fitting

In order to reduce the sensitivity to surface irregularities that could arbitrarily increase the surface area and decrease the accuracy of the above descriptors, a 3D ellipsoid can be fitted to the object. The fitting of the ellipsoid is performed using the classical moment-based procedure. Elongation and flatness are the ratios between the different radii of the fitted 3D ellipsoid.

(2) $$\text{Elongation}=\frac{R_{1}}{R_{2}},\text{Flatness}=\frac{R_{2}}{R_{3}}$$

where *R*_1_, *R*_2_ and *R*_3_ are three radii of the fitted ellipsoid, in decreasing order.

The ratio between the best fitted 3D ellipsoid volume and the actual volume of the object can also give a good idea of how far the object is from an ellipsoid.

(3) $$\text{Ratio}=\frac{\frac{4}{3}\pi R_{1}R_{2}R_{3}}{V}$$

#### 3D geometric moments

These six shape descriptors are invariant with respect to change of scale, translation and rotation. They are based on central moments up to the fourth order, and describe the variations of the object from the ellipsoid. They are described in detail in [18].

### Classification-based segmentation

**Figure 2:**
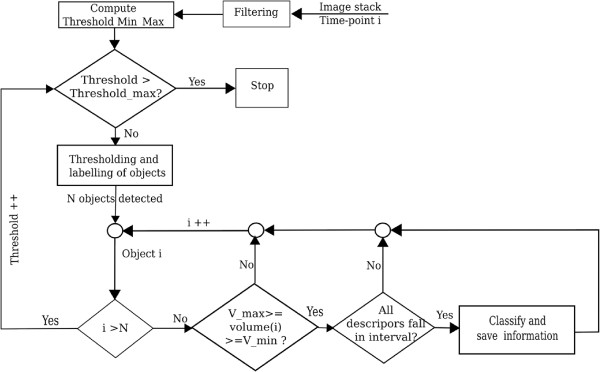
**Flowchart for building the hierarchy of objects used by the segmentation and classification procedure.** When an object is well classified its information is saved and the object is inserted into the hierarchy structure.

**Figure 3:**
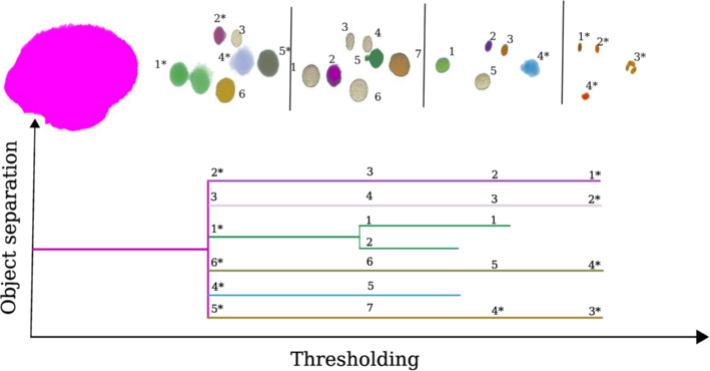
**Hierarchy structure of classified objects.** Starting with a low threshold value, the whole embryo is segmented but cannot be classified as a valid object. When the threshold value increases, objects can be classified as valid and put in the hierarchy with the saved information. In the case of object separation, the associated object at the previous threshold, if any, is computed in order to put the objects in their right place in the hierarchy. The best object inside each branch is computed by maximizing the classification, and the segmented object is reconstructed.

After the training, features are extracted from the nuclei that are validated by the user, the main classification-based segmentation is applied. As mentioned above, the procedure is based on an iterative thresholding method. For a given time point, the 3D filtered image is first segmented using a low threshold and all detected objects are extracted. For each detected object, we first check if the volume falls into the interval of volumes for all classes. If the volume of the object is valid, each descriptor value is compared against the minimum and maximum descriptor values for all classes. Finally, if and only if the object has a valid volume and descriptor values, it is classified using the trained classifier and the corresponding object information (current threshold, classification results, volume, coordinates, etc.) is saved (Figure 2). Since the procedure starts with a low threshold, the whole embryo would be considered as one object, but as a result of this value checking, it will not fall into the interval of accepted volumes and will therefore not be classified, and the next threshold will be used. As the threshold increases, nuclei start to emerge from the embryo. They can be merged as one object for a low threshold, but they will be separated with higher thresholds (Figure 3). With increasing threshold values, objects change in shape and may therefore be classified into different classes. Once the thresholding terminates, we obtain a hierarchy of objects that shows the separation of nuclei with increasing thresholds (Figure 3).

In order to find the best threshold in each branch of this hierarchy (*i.e.*, for each object), we search for the threshold values that stabilize the shape of the object and, therefore, the associated class. More precisely, for each branch, starting from the right (*i.e.*, high thresholds), we look for the largest interval with consecutive classification in the same class. The threshold that provides the highest probability for this class is kept. The object in the branch is then reconstructed using the associated threshold.

### Using *a priori* biological knowledge

In our procedure, branches with higher thresholds are favored. This provides good separation of nuclei, even when they are close (as in anaphase).

In some cases, like in our *C.elegans* dataset, some prophase nuclei display very condensed chromosomes that look similar to metaphase nuclei. Due to our strategy, whole nuclei will be segmented using low thresholds and classified as prophase. However, for higher thresholds, condensed chromosomes inside may be segmented and classified as either prophase or metaphase. Therefore, we implemented a generic inclusion model that does not allow particular classes (prophase, in this case) to include other classes (prophase and metaphase).

## Results

All figures were done with FigureJ [19].

### Datasets

The algorithm was applied to three different datasets: the first two were acquired from two different biological models and the third one was a synthetic dataset that simulated dividing cells (Table 1). Our original motivation was to automate the segmentation of nuclei in the *C. elegans* dataset where none of the available methods had provided satisfactory results. The *Drosophila* dataset was obtained from an existing reference and hence represents an additional validation dataset [6]. In their study, the authors used a Histone-GFP labeling method with confocal microscopy. For our *C. elegans* dataset, a slightly different labeling procedure was used, with Histone-GFP expressed in the ovary and transmitted to the future embryo. The fluorescence signal was thus gradually diluted with mitosis, leading to an overall decay in fluorescence intensity and, consequently, of image quality over time. Another difference between the two datasets was that *Drosophila* embryos were imaged with a conventional confocal microscope [6], whereas *C. elegans* embryos were imaged with a spinning disk microscope. The third dataset was a synthetic dataset generated using CytoPack [20], a program that models dividing cells and creates synthetic datasets that simulate 4D video-microscopy acquisition. Although the aspect of nuclei was realistic, it was not completely natural, and could present high intensity variation, thus providing a difficult test case for our algorithm.

**Table 1:** Origin and characteristics of the three datasets

**Model**	**Acquisition**	**Size (XYZT)**	**Calibration (xy-z)**	**Stage**	**Time interval**	**Reference**
*C. elegans*	Spinning disk	512×512×31×160	125×350 nm	90	1 min	This paper
*Drosophila*	Confocal	1024×1024×68×30	130×440 nm	330	2 min	[6]
Simulated	‘Video’	512×512×59×76	125×200 nm	30	2 min	[11]

Stage refers to the number of nuclei at the end time point.

### Supervised learning

Before using the classifier in the main classification-based segmentationtion algorithm, we had to validate it. For this purpose a first test was performed using objects segmented by the procedure presented in the *Supervised learning* section. This procedure is based on a MSER algorithm and uses the volumes given by the user as inputs. For each dataset, between 100 and 200 sample nuclei, with approximately equal number for each class, were chosen from different time points according to shape variability, and then assigned a class by a human expert. For all of the datasets, the confusion matrix for the classifier showed satisfactory results, with a classification rate of approximately 90% for the two real embryo datasets and 80% for the synthetic dataset (Table 2).

**Table 2:** Classification results for the set-up and validation of the classifier

***C. elegans***						***Drosophila***						**Synthetic**					
	**I**	**P**	**M**	**A**	**T**		**I**	**P**	**M**	**A**	**T**		**I**	**P**	**M**	**A**	**T**
Inter	13	2	0	0	0	Inter	11	0	0	0	0	Inter	17	0	0	0	0
Pro	4	20	0	0	0	Pro	0	8	0	0	0	Pro	1	4	1	1	0
Meta	0	1	12	0	0	Meta	0	1	9	0	0	Meta	0	0	3	0	0
Ana	0	0	0	17	0	Ana	0	1	0	5	2	Ana	1	1	0	4	0
Telo	0	0	0	1	11	Telo	0	0	0	1	6	Telo	0	1	0	2	0

The number of samples used were 243, 128 and 106 samples for *C. elegans, Drosophila*, and synthetic data, respectively. The classification accuracies were 90%, 89% and 78%, respectively.

### Segmentation

The efficiency and accuracy of the proposed algorithm was tested on the three complete datasets (see Table 3). The segmentation results were visually compared with original data for all time points and taking the 3D information into account. We used a classification based on five classes (interphase, prophase, metaphase, anaphase and telophase) for all three datasets. Between 10 and 30 sample nuclei per class were chosen for the initialization (supervised training of the classifier). The values used as volume limits for the *C. elegans* dataset were (in *μm*^3^): 9.8–24.3 (interphase), 14–36.6 (prophase), 5.9–19 (metaphase), 3.6–7.3 (anaphase) and 6.3–9.9 (telophase). For the *Drosophila* dataset, the minimum and maximum volumes were set to (in *μm*^3^): 45–100, 120–150, 36–40, 25-39 and 20–28.

**Table 3:** Segmentation results

**Model**	**Time**	**TP**	**FN**	**FP**	**Recall**	**Precision**	**F-measure**
*C. elegans*	T86	43	1	0	0.977	1	0.988
*C. elegans*	T122	63	3	0	0.95	1	0.979
*C. elegans*	Total (1–128)	3585	21	2	0.9923	0.9997	0.9960
*Drosophila*	T5	98	0	3	1	0.97	0.985
*Drosophila*	T9	172	0	3	1	0.983	0.99
*Drosophila*	T25	254	3	3	0.988	0.988	0.988
*Drosophila*	Total (1–30)	4820	15	77	0.9969	0.9843	0.9905
Synthetic	Total (1–76)	1974	0	0	1	1	1

TP = True Positive (ground truth number of objects). FN = False Negative (non detected). FP = False Positive (noisy detected). Recall = TP/(TP + FN). Precision = TP/(TP + FP). F = 2*precision*recall/(precision + recall).

**Figure 4:**
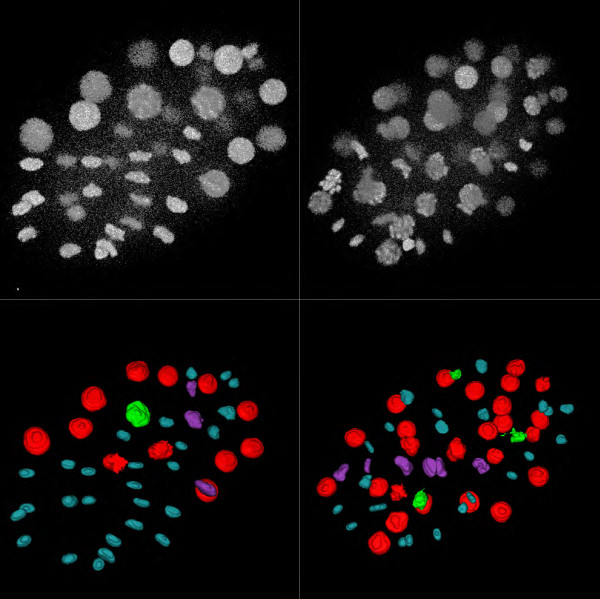
**Results of segmentation and classification on the *C. elegans *dataset.** Top row: 3D view of segmented data for two different time points (T = 86,122). Bottom row: 3D view of classified data for the same time points. Note that all objects are well separated.

**Figure 5:**
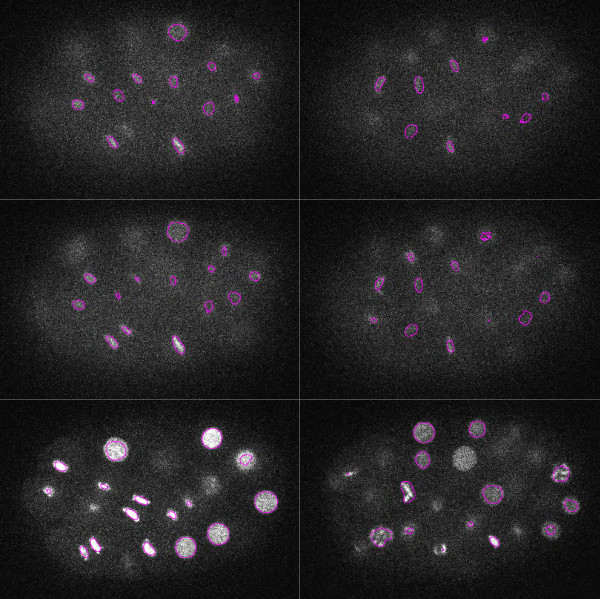
**Segmentation contours overlaid on different slices (Z = 8,9,21) of raw data for the *C. elegans * dataset.** Left and right columns correspond to left and right columns of Figure 4. Note that even hard to distinguish nuclei could be detected.

For the *C. elegans* dataset, there was a decay of intensity with time due to the particular labeling technique and acquisition system, and the image quality was therefore not as high as in the *Drosophila* dataset. Furthermore, due to the embryo depth and imaging conditions, the slices farthest away from the objective lens were quite noisy. The selected thresholds corresponding to segmented nuclei varied between time points as well as within each time point image. The threshold values varied between 883 and 1,757 (with an average of 1,180) for time point 40, between 754 and 1,200 (with an average of 900) for time point 86, and between 652 and 1,064 (with an average of 781) for time point 122. However almost all of the nuclei could be correctly detected (correct position) for all time points (Additional file 1). Nevertheless, after time point 128, the image quality was too low to accurately delineate the actual shape of the nuclei. Figure 4 shows the results obtained for some of the time points. At an advanced time point (122), all nuclei were correctly segmented despite the low quality of the image (Figure 5). For the time points to 128, only 21 out of 3,585 (0.3%) were not detected, with zero false negatives.

**Figure 6:**
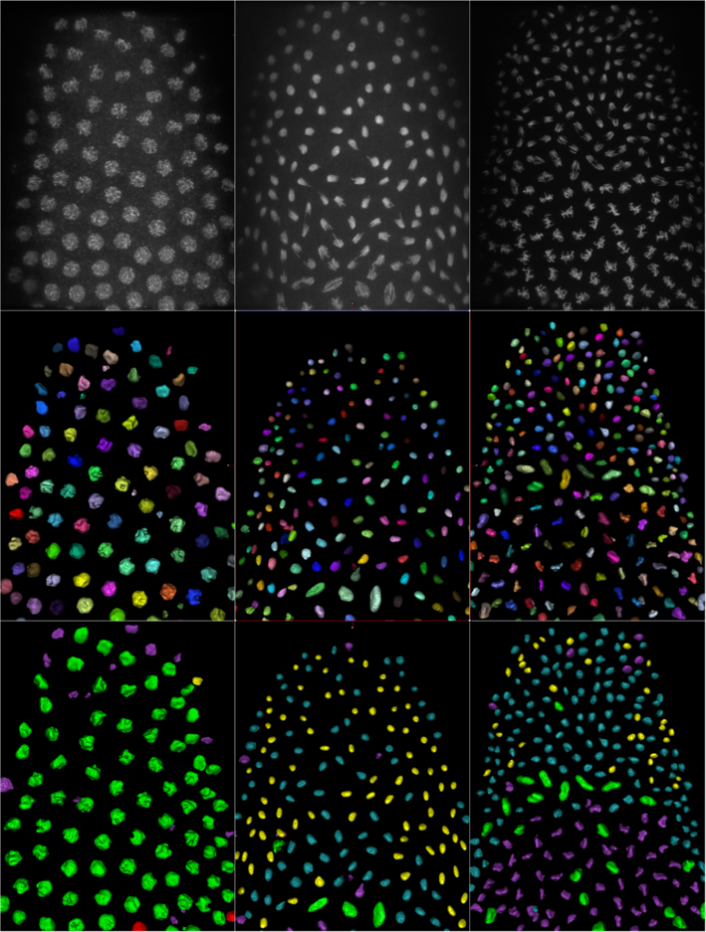
**Results of segmentation and classification on the *Drosophila *dataset for different time points (T = 5,9,25).** Top row : 3D view of original raw data. Middle row : 3D view of segmented data. Bottom row : classification results (Red = interphase, Green = prophase, Magenta = metaphase, Cyan = anaphase, Yellow = telophase). Note that in bottom middle, almost all of the nuclei have just divided; they are in anaphase or telophase stages and are correctly segmented and classified.

In the *Drosophila* dataset, which has a higher signal quality, nuclei were generally present in a 2D layer. All nuclei were correctly segmented, only 15 out of 4,820 (0.3%) were not detected, and an additional 77 spurious noisy objects were incorrectly identified as nuclei (1.6%) (Figure 6) (Additional file 2). The selected threshold values did not vary much, which was coherent with the homogeneous intensity throughout the entire 4D dataset. The difference between the lowest and highest threshold value used was 28 for time point 5, 34 for time point 9, and 36 for time point 25.

For the simulated dataset, all synthetic nuclei were correctly segmented despite high intensity variance within nuclei (Additional file 3).

**Figure 7:**
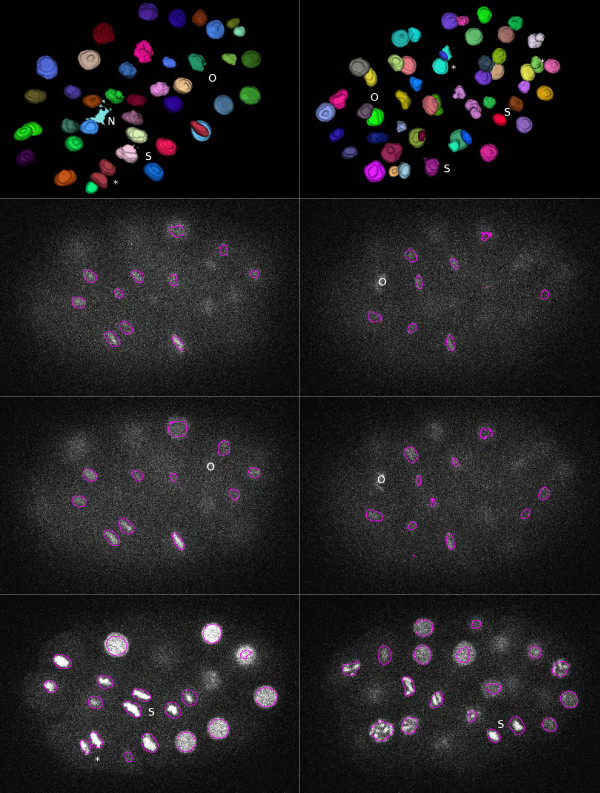
**Results of segmentation on the *C. elegans *dataset with the HK-Means method for the same time points and the same z slices as Figures **5**and **6**.** Top row: 3D surface view of segmented objects. Next rows: some slices with contours overlaid. (*) indicates merged nuclei; (N) indicates noisy detected object, (O) indicates missing object; and (S) indicates inaccurate segmentation, especially for metaphase nuclei.

The results obtained with our method on *C. elegans* data (our target dataset) were compared with the Hierarchical K-Means method (HK-Means) [21] of ICY [22]. This method implements a slightly modified version of the MSER technique and is therefore probably one the best available methods to compare to. With the same volume parameters as for our algorithm, HK-Means was globally less accurate. Several detected objects were actually noise, some nuclei were not detected, and several others were merged. For example, at time point 86, there was one noisy object, three nuclei were missing, and there was one fusion of two nuclei; for time point 122, there were six noisy objects, two nuclei were missing, and two were merged (Figure 7). Since HK-Means method favors low threshold values, the objects tended to be larger, and the delineation of nuclei was globally not as accurate as with our algorithm.

The time to segment and classify 128 time points for the *C. elegans* dataset (160 frames, 512×512×31 voxels) on a Xeon bi-processor with 12 cores/24 threads (Xeon X5660@2.80 Ghz), running Ubuntu Linux 12.04 with 24 Gb of RAM and using Java 1.7, was between 4 and 6 hours, corresponding to approximately 3 minutes per time point.

### Classification

Although the purpose of the classification procedure in our method is related to the selection of the best threshold value for segmentation, it can also be used collaterally as a classification algorithm *per se* to predict cell-cycle phases. The information about the cell-cycle stage given by the algorithm can be used for cell-cycle analysis and to help further tracking. We visually analyzed the classification results on a frame-by-frame basis, using temporal information to determine the actual cell-cycle stage.

For the *C. elegans* dataset, 165 out of 3,583 nuclei (for time points 1–128) were not correctly classified (4.6%), and classification errors occurred uniformly in all time points. This error rate was less than the training error (∼10%, see Table 1) because of the high number of interphase nuclei in the entire dataset, and mainly resulted from shape similarity between classes, especially at the anaphase and telophase stages.

For the *Drosophila* dataset, 225 out of 4,820 nuclei were not correctly classified (4.7%). 186 of these classification errors occurred in the three last time points. This error rate was similar to the one previously reported [23].

For the synthetic dataset, where all nuclei were correctly segmented, the classification results were similar, with 96% of correctly classified nuclei. Due to the non-natural aspect of the simulated nuclei, the distinction between some stages was not obvious: the prophase and telophase stages were difficult to discriminate from other stages, whereas the interphase, metaphase and anaphase stages were correctly classified (Additional file 3).

## Discussion and conclusion

We have presented a machine-learning based approach for segmenting nuclei in 3D microscopy images. The originality of the approach is to perform segmentation and classification simultaneously within an iterative threshold selection procedure, ensuring that the best threshold for each object will be found. By comparing our approach with HK-Means, we evaluated the benefit of using a machine-learning approach to improve the segmentation accuracy by detecting objects similar in shape to training samples. The segmentation provided by our approach is more accurate because it is the one that leads to the best classification score. From the point of view of classification, we introduced with the paradigm of “jointly segment and classify” rather than “segment then classify”, a new idea that could be used as a basis for further algorithms.

The major advantage of our method is its ability to robustly adapt to different imaging conditions and different species by reporting the introduction of the required knowledge on the initial learning stage from user-specified samples. The only assumption that is made is that an object can be segmented by one threshold value and has a class-specific shape.

Our algorithm was successfully tested on two embryo models, the *C. elegans* dataset (our work dataset), and the *Drosophila* dataset (our validation dataset). The overall segmentation results were comparable or better than those of existing algorithms, without the necessity of fine-tuning parameters. The only required parameters are the volumes of nuclei that can be easily learned from a set of trials and errors at the supervised learning step, where all descriptors values are displayed to the user. The user can take some time in training the system but it only needs to be done once per embryo model and imaging conditions. The same set of training descriptors can be re-used in different experiments. The implemented 3D descriptors summarize the shape of the object and do not take quantitative variable aspects such as object volume, surface or intensity into account since these values can be extremely variable over time. In fact, in the first time points of the series, nuclei are usually very large and very bright, contrasting with the numerous darker small nuclei at end time points.

For the *Drosophila* validation dataset, only a few nuclei were not detected (0.3%), and generally corresponded to very crowded telophase nuclei that displayed very unusual shapes. However, it seems that we detected more nuclei than the original paper [6] (4,820 compared to 4,166; it is possible that some border objects were not counted). We were also able to achieve an overall acceptable classification with an error rate of less than 5%, where classification errors were mainly present at end time points for very crowded interphase nuclei.

For our working dataset, *C. elegans*, the results for segmentation and classification were quite satisfactory, even in a context of low signal-to-noise ratio. We can then validate the acquisition and labeling procedure for further studies to improve automated lineage reconstruction.

For the synthetic dataset, we observed significantly better classification results in the entire data than in the training samples because of the over-abundant interphase nuclei (1,586 out of 1,974), which were easier to classify than the other ones.

Classification is a very important step in our thresholding procedure. However, in order not to add a threshold parameter to the classification results, we preferred to add an interval check of volumes and of all descriptor values prior to classification, acting as a Boolean pre-classifier. Nevertheless the classification error rate was quite low, although it is not an easy task, even for an experienced biologist, to distinguish between the five cell-cycle stages we defined. With only three or four classes (merging anaphase and telophase, for example) we would expect higher classification results. Furthermore, since interactive selection of samples for the supervised learning phase is an essential part of the method, we implemented a semi-automatic procedure to make this task easier. The user may have a tendency to choose rather non-ambiguous nuclei in the different cell-cycle stages. However, the nuclei in the different stages present continuous variations that lead to small changes in shape, thus inducing misclassification. For tracking analysis, this misclassification could be alleviated by taking the class of the object at the previous time point into account. Some noisy objects were also detected, but they can be easily removed by post-processing procedures, as they appeared for some threshold values only, presented peculiar shapes or intensities, and had low classification scores. They could be easily filtered out using available tools such as the 3DRoiManager [24,25]. Since we did not want to add extra parameters, we chose to keep these noisy objects and just warn the user about them with a screen message.

The algorithm may be quite time-consuming since many threshold values are tested, especially in 16-bits. However, the user can speed it up by increasing the step between two thresholds or by converting the data to 8-bits. Furthermore, using multi-threaded algorithms speeded up the process, leading to less than 3 minutes to process one time point.

Our results suggest that the segmentation method proposed can also be applied to other images and provides a promising start for tracking analysis using classification information.

## Competing interests

The authors declare that they have no competing interests.

## Authors’ contributions

JGM implemented the segmentation algorithm; IAC implemented the classification; PA participated in the design of the study; VG provided the *C. elegans* data, participated in the design of the study and validated the results; TB implemented the 3D descriptors and designed the study; JGM, IAC and TB analyzed the results; JGM, IAC, PA and TB wrote the paper. All authors read and approved the final manuscript.

## Supplementary Material

Additional file 1 **Movie 1a.** 4D view of raw data for the C. elegans dataset. **Movie 1b.** 4D view of segmented data with classification for the C. elegans dataset. **Movie 1c.** Movie with contours overlaid on raw data for time point 122 for C. elegans. Click here for file

Additional file 2 **Movie 2a.** 4D view of raw data with for the Drosophila dataset. **Movie 2b.** 4D view of segmented data with classification for the Drosophila dataset. **Movie 2c.** Movie with contours overlaid on raw data for time point 25 for Drosophila. Click here for file

Additional file 3 **Movie 3a.** 4D view of raw data for the simulated dataset. **Movie 3b.** 4D view of segmented data with classification for the simulated dataset. Click here for file
